# Nano-Scale Alignment of Proteins on a Flexible DNA Backbone

**DOI:** 10.1371/journal.pone.0052534

**Published:** 2012-12-26

**Authors:** Tatsuya Nojima, Hiroki Konno, Noriyuki Kodera, Kohji Seio, Hideki Taguchi, Masasuke Yoshida

**Affiliations:** 1 Department of Molecular Biosciences, Kyoto Sangyo University, Kyoto, Japan; 2 Bio-AFM Frontier Research Center, Institute of Science and Engineering, Kanazawa University, Kanazawa, Japan; 3 Department of Life Science, Tokyo Institute of Technology, Yokohama, Japan; 4 Department of Biomolecular Engineering, Tokyo Institute of Technology, Yokohama, Japan; University of Helsinki, Finland

## Abstract

Nano-scale alignment of several proteins with freedom of motion is equivalent to an enormous increase in effective local concentration of proteins and will enable otherwise impossible weak and/or cooperative associations between them or with their ligands. For this purpose, a DNA backbone made of six oligodeoxynucleotide (ODN) chains is designed in which five double-stranded segments are connected by four single-stranded flexible linkers. A desired protein with an introduced cysteine is connected covalently to the 5′-end of azido-ODN by catalyst-free click chemistry. Then, six protein-ODN conjugates are assembled with their complementary nucleotide sequences into a single multi-protein-DNA complex, and six proteins are aligned along the DNA backbone. Flexible alignment of proteins is directly observed by high-speed AFM imaging, and association of proteins with weak interaction is demonstrated by fluorescence resonance energy transfer between aligned proteins.

## Introduction

Protein-protein interactions play a critical role in numerous biological processes, and understanding the nature of each interaction is of central importance in biology and biotechnology. When the interactions are strong, proteins associate into a stable homo- or hetero-oligomer complex that can be isolated relatively easily. In many cases, however, interactions are transient or weak, producing a complex that is difficult to isolate. To study such complex, component proteins of the complex are chemically cross-linked or genetically fused as a single polypeptide to prevent the complex from dissociation. However, these methods have some limitations. Chemical cross-linking has a limited reactivity and is not easy to control the number of cross-linked proteins. Fused protein has structural restriction and is often difficult to express in a soluble form. For effective formation of complex, proteins should be linked with high flexibility enough to allow proteins to move and turn freely to associate with each other. Here we report a procedure to align several different proteins, all connected to a single flexible DNA backbone. This increases local concentrations of the proteins and will enable association of proteins with very weak interactions.

## Materials and Methods

### Protein Preparation

Expression plasmid pET21c-His_6_-sfGFP-Cys was generated from pET21c-wild-type GFP by site-directed mutagenesis using the PrimeSTAR mutagenesis basal kit (Takara, Japan). We used Cerulean [1] and Venus [2] as improved CFP and YFP. A cysteine residue was introduced at the N-terminus of CFP (Cys-CFP) and at the 173 position of YFP (Cys-YFP), taking into consideration their orientations in the dimer [3]. A206K mutations (monomer propensity) and S208F/V224L mutations (dimer propensity) were also introduced. Proteins were expressed in *Escherichia coli* BL21(DE3) at 20°C for 20 hr. Harvested cells were sonicated in buffer (20 mM Tris-HCl, pH 7.4, 1 mM DTT, a Complete protease inhibitor mixture tablet (Roche Applied Science)) and centrifuged. In the case of sfGFP purification, cell lysate was heat-treated at 80°C for 15 min before centrifugation. The supernatant was applied to anion exchange column (DEAE-650M TOYOPEARL), eluted by step-wise increase of NaCl concentration (0–100 mM), and subsequently purified by Ni-sepharose column (GE healthcare).

### Azido-ODN (N_3_-ODN) Preparation

5′-NH_2_-(CH_2_)_6_-ODNs were synthesized by an automated DNA synthesizer using standard phosphoramidite chemistry. The lyophilized DNA was dissolved in the buffer (100 mM Bicine-KOH, pH8.0, 1 mM EDTA) at the concentration of 100 µM. Azide-PEG_4_-NHS ester (160 mM) (Click Chemistry Tools, USA) in DMSO was added (final concentration, 5 mM) to the DNA solution and reacted for 2 hr at 37°C. The solution was applied to an anion exchange column (DEAE-650M TOYOPEARL) equilibrated with buffer (50 mM Tris-HCl, pH7.4, 50 mM NaCl). The column was washed three times with six-column volume of buffer (50 mM Tris-HCl, pH7.4, 50 mM NaCl) to remove the unreacted azide-PEG4-NHS ester. N_3_-ODN was subsequently eluted by 500 mM NaCl. The purified N_3_-ODN was desalted by ethanol-precipitation, dissolved in TE buffer (20 mM Tris-HCl, pH7.4, 1 mM EDTA) and stored at −80°C.

### sfGFP-ODN Preparation by Strain-promoted Azide-alkyne Catalyst-free Click Chemistry

The reaction mixture containing 20 µM His_6_-sfGFP-Cys, 20 µM DBCO-PEG_4_-Maleimide (Click Chemistry Tools, USA), 40 µM N_3_-ODN in buffer (20 mM Tris-HCl, pH7.4, and 100 mM NaCl) was incubated at 37°C for 10 hours. Yield of the sfGFP-ODN production was analyzed by SDS-PAGE. To remove remaining free protein, the reaction mixture was applied to an anion exchange column (DEAE-650M TOYOPEARL). sfGFP-ODN has negative charges due to the phosphate backbone of DNA and has higher affinity to the anion exchange column than does free protein. The column was washed with a low-salt buffer (20 mM Tris-HCl, pH7.4, 100 mM NaCl) and sfGFP-ODN was eluted by a high-salt buffer (20 mM Tris-HCl, pH7.4, 500 mM NaCl). The eluted solution was applied to a Ni-column (Ni-sepharose, GE healthcare) to remove the unreacted N_3_-ODN. The column was washed with the low salt buffer and removal of unreacted N_3_-ODN was monitored by absorbance of at 280 nm. sfGFP-ODN was eluted by the low salt buffer supplemented with 400 mM imidazole.

### Formation of 5dsDNA-backbone and Multi-protein-DNA Complex

Six kinds of ODNs listed in table 1 or six kinds of sfGFP-ODNs made from these ODNs were mixed at the final concentration of 100 nM in 50 mM Tris-HCl, pH7.4 and 100 mM NaCl, and incubated at 37°C for 1 hour. The formation of multi-protein-DNA complex was confirmed by Native PAGE (8%) in which GFP fluorescence was detected by ImageQuant LAS-4000 (Fuji-Film, Japan).

**Table 1:** Sequence of ODNs used in this study. Primary amine (NH2) was attached to 5′-end via (CH_2_)_6_ spacer. Underlines indicate restriction enzyme sites.

No.-(length)	Sequence
1-(55 nt)	5′NH_2_-(CH_2_)_6_-CGATTGTGGTAGTGAATTCGTCCAGGTTTCTCTTAGAGGATCCAGTGAGCGTATC3′
2-(55 nt)	5′NH_2_-(CH_2_)_6_-GATTACGATATTGCCCGGGTGTGTCATTTCGCTAGATCTCGAGGAGTCCGCAAAG3′
3-(26 nt)	5′NH_2_-(CH_2_)_6_-GTTGTGAGTGAAGCTTAGCCATGATG3′
4-(55 nt)	5′NH_2_-(CH_2_)_6_-CATCATGGCTAAGCTTCACTCACAACTTTCTTTGCGGACTCCTCGAGATCTAGCG3′
5-(55 nt)	5′NH_2_-(CH_2_)_6_-TGACACACCCGGGCAATATCGTAATCTTTGATACGCTCACTGGATCCTCTAAGAG3′
6-(26 nt)	5′NH_2_-(CH_2_)_6_-CCTGGACGAATTCACTACCACAATCG3′

### High-speed Atomic Force Microscopy

To observe the molecular shapes of the 5dsDNA-backbone and multi-protein-DNA complex, we performed high-speed AFM imaging in the tapping mode using a laboratory-built apparatus [4], [5] and small cantilevers (Olympus) with a spring constant of 0.1–0.2 N/m and a resonant frequency of 0.8–1.2 MHz in buffer solution. Diluted samples (3–5 nM) of 5dsDNA-backbone and multi-protein-DNA complex in buffer A (10 mM Tris-HCl, pH 7.4, 2 mM MgCl_2_) were deposited on an APTES-mica surface [6] and on a freshly cleaved mica surface for 3 min, respectively. To remove unattached molecules, the sample surface was rinsed with buffer A (∼20 µL) without drying. Then, the sample surface was immersed in a liquid cell filled with buffer A (∼60 µL). In the liquid cell, a small cantilever was fixed. Then, high-speed AFM imaging was performed in buffer A. Here, the diluted samples were used within 3 hours.

### FRET Measurement

Cys-CFP conjugated with ODN-4 (55 nt) and ODN-6 (26 nt) and Cys-YFP conjugated with ODN-1 (55 nt) were prepared as above. For preparation of a protein-DNA complex in which two proteins are at neighboring positions, 400 µl of the solution containing 50 nM CFP-ODN-6 and YFP-ODN-1 in buffer (50 mM Tris-HCl, pH7.5, 10 mM MgCl_2_, 1 mM dithiothreitol, 100 mM NaCl and 0.01% (w/v) bovine serum albumin) was prepared. For preparation of a protein-DNA complex in which two proteins are at opposite end positions, 400 µl of the solution containing 50 nM CFP-ODN-4 (55 nt), YFP-ODN-1 (55 nt) and other four ODNs (−2 (55 nt), −3 (26 nt), −5 (55 nt) and −6 (26 nt)) was prepared. Solution was incubated at 37°C for 1 hour, divided in two halves, and one half was treated with 15 units of EcoRI (Takara, japan) at 37°C for 1 hour. Then fluorescence spectrum was measured (excitation 430 nm/emission 450–600 nm, FP-6500 (Jasco, JAPAN)).

## Results

**Figure 1:**
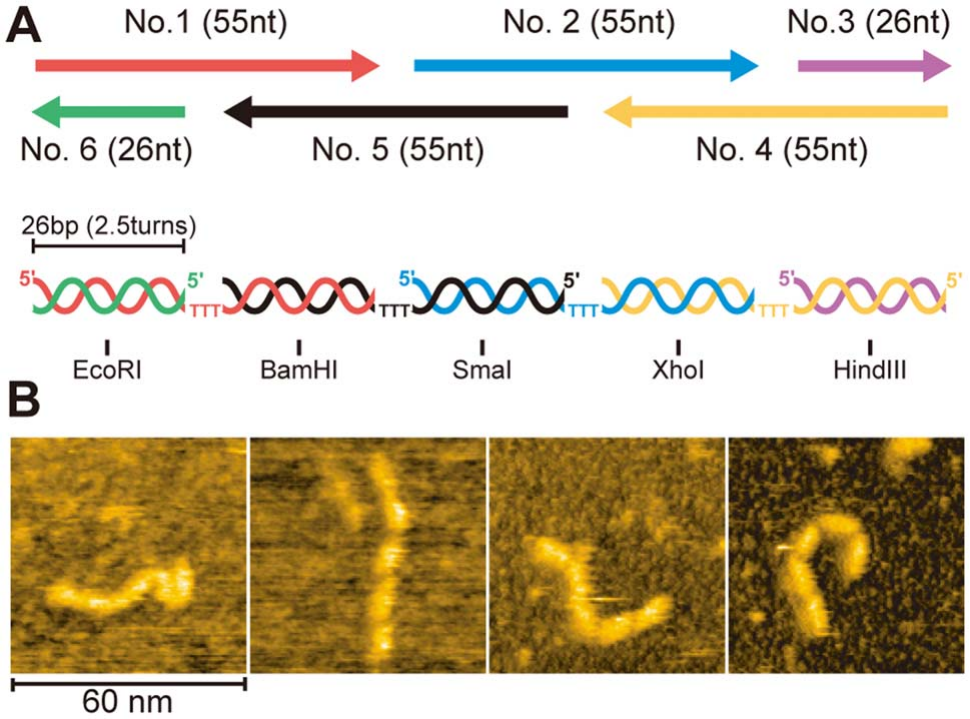
Flexible DNA backbone. (A) Hybridization of four 55 nt ODNs (numbered 1, 2, 4 and 5) and two 26 nt ODNs (numbered 3 and 6). Five 26 bp dsDNA segments are connected by ssDNA (three thymines). The restriction sites are also shown. (B) AFM images of flexible DNA backbone.

We used DNA structure as a backbone for the designed alignment of several proteins. DNA is widely used to construct nano-architecture by taking advantage of its specific recognition of complementary nucleotide sequences. Recently, enzymes have been aligned on a DNA scaffold to facilitate consecutive reactions [7]–[14]. The DNA scaffolds in these works are rigid and proteins on the scaffold cannot associate with each other. For our purposes, we designed a DNA backbone composed of five solid segments, each connected by a flexible linker (Fig. 1A). The designed DNA backbone consists of six ODNs, of which two have a length of 26 nt and four have a length of 55 nt. For effective hybridization at temperatures safe for proteins, the nucleotide sequences with least propensity for making secondary structure were chosen using the NUPAC web server (http://www.nupack.org/) [15] (table 1). The hybridized structure contains five double-stranded DNA (dsDNA) segments connected by four single-stranded trithymidylate regions. The length of all dsDNA segments is 26 bp, which is approximately equal to 2.5 turns of the helices (8.4 nm), so the 5′-end of the ODNs in each dsDNA segment is oriented to the same direction. The single-stranded portions (∼1.0 nm) are flexible and enable the five dsDNA segments to take different orientations. A unique restriction enzyme sequence was introduced in each dsDNA segment for future application. The hybridized product of six ODNs (5ds-DNA backbone) was imaged by high-speed AFM [4], [5] (Fig. 1B). As expected, five solid bars (corresponding to five dsDNA segments) are connected in tandem like a train with five cars. Linkers between bars are flexible, and the 5ds-DNA backbone adopted various overall shapes. The observed contour length of 5ds-DNA backbone (∼50 nm) agrees with the predicted length (∼46 nm).

**Figure 2:**
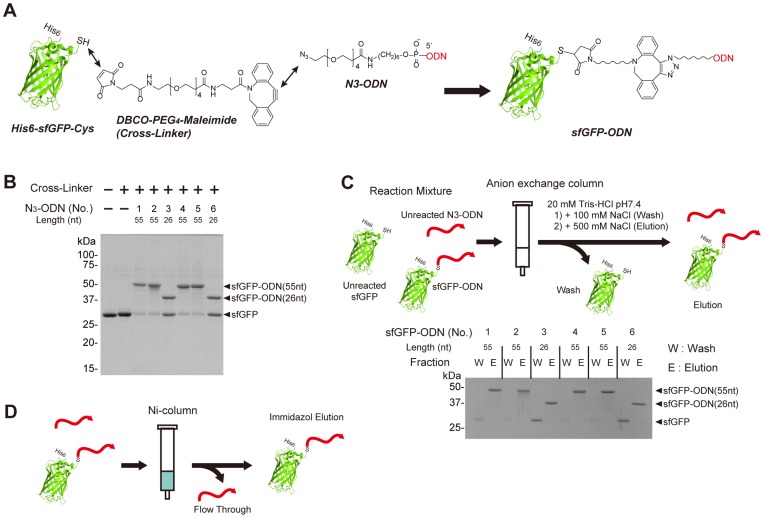
Formation of sfGFP-ODN. (A) Cysteine-introduced sfGFP (His_6_-sfGFP-Cys) and N_3_-ODN was conjugated via DBCO-PEG_4_-maleimide. (B) Formation of sfGFP-ODN was analyzed by SDS-PAGE. Proteins in the gel were stained and shown. (C) Purification of sfGFP-ODN. The reaction mixture was applied to an anion exchange column. Free sfGFP was washed out by 100 mM NaCl, and sfGFP-ODN was eluted by 500 mM NaCl. “Wash” and “Elution” fractions were analyzed by SDS-PAGE. (D) Removal of unreacted ODN. The solution was applied to Ni-column. Only sfGFP-ODN was captured on the column by hexa-histidine tag of sfGFP and unreacted ODN was removed. sfGFP-ODN was eluted by imidazol.

Conjugation of proteins and ODNs by using a hetero-bifunctional cross-linker with NHS ester and maleimide has already been reported [16], [17]. We adopted strain-promoted azide-alkyne catalyst-free click chemistry [18] because of its storage stability and reaction specificity (Fig. 2A). 5′-aimino-ODNs were reacted with azide-PEG_4_-NHS ester and azido-ODNs (N_3_-ODNs) were synthesized. Unreacted excess aizde-PEG_4_-NHS was removed by anion-exchange column. Synthesis of N_3_-ODN was confirmed by MALDI-TOF MS (Fig. S1). Because this reaction proceeds efficiently and unreacted 5′-amino-ODN does not participate in next reaction, further purification is not required. We confirmed that the N_3_-ODN is stable for several months at −80°C. “Superfolder green fluorescent protein [19]” (sfGFP) was used as a model protein because of its stability and easy detection by fluorescence. A hexa-histidine-tag was attached to the N-terminus for easy purification, and an extra cysteine residue was attached at the C-terminus of sfGFP for the next crosslinking reaction. A maleimide-introduced aza-dibenzocyclooctyne (DBCO-PEG_4_-Maleimide) was used as a bifunctional cross-linker between the cysteine residue and azide. In SDS-PAGE analysis, the conjugate of sfGFP and ODN (sfGFP-ODN) appeared at a position of higher molecular weight than unmodified sfGFP (Fig. 2B). When an extra cysteine residue was not attached at C-terminus of sfGFP, sfGFP and ODN were not conjugated (Fig. S2). sfGFP-ODN has additional negative charges derived from phosphate backbone of DNA and unreacted free sfGFP was easily removed from the solution by anion-exchange column (Fig. 2C). sfGFP-ODN was adsorbed to metal-chelating affinity resins (Ni-column) through its hexa-histidine tag and was separated from unreacted N_3_-ODN (Fig. 2D). We confirmed that the sfGFP and ODN were conjugatedas designed using mass spectroscopy (Fig. S3).

**Figure 3:**
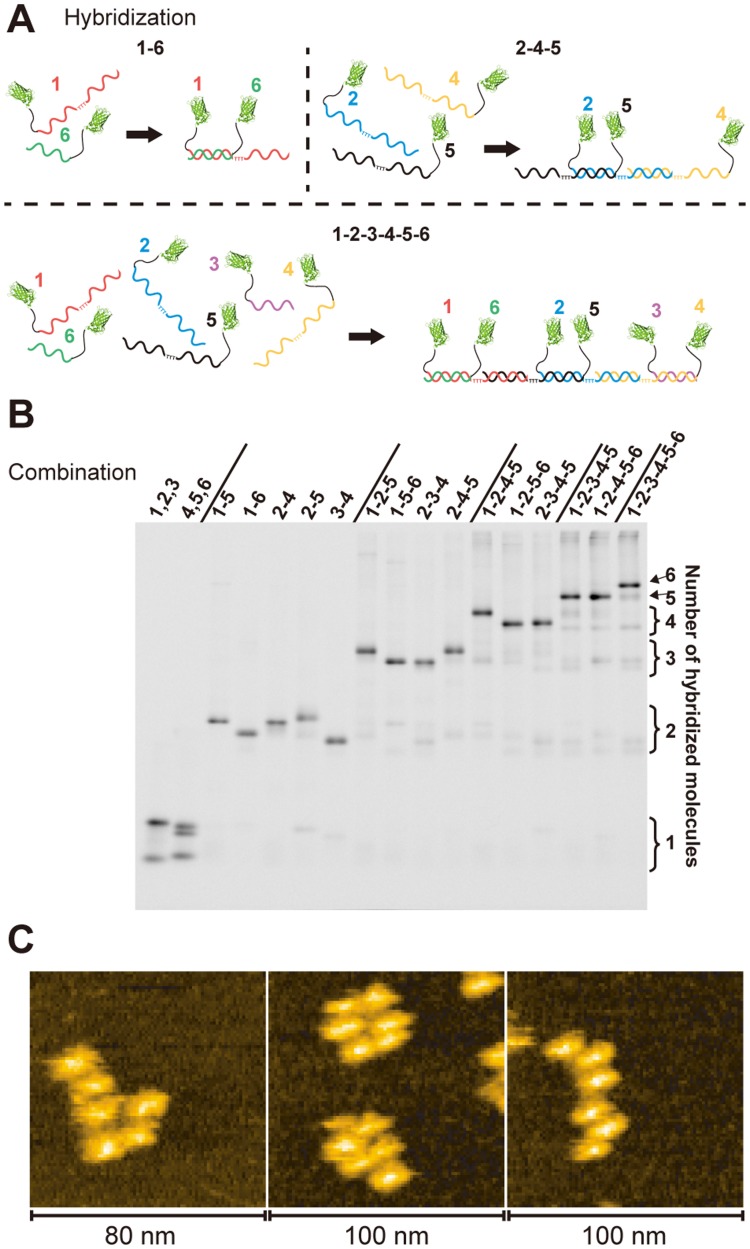
Formation of multi-protein-DNA complex. (A) Three representative examples of hybridization of sfGFP-ODNs are shown (B) Various combinations of sfGFP-ODNs, as shown by numbers on top of the lanes, were mixed, hybridized and analyzed by Native-PAGE. GFP fluorescence in the gel was detected. No hybridization occurred for the two leftmost combinations. The rightmost lane shows the products of six sfGFP-ODNs. (C) AFM images of the hybridized product containing six sfGFP-ODNs. Various arrangements of the six concatenated particles were observed.

Various combinations of purified sfGFP-ODNs were mixed (Fig. 3A) and the products of hybridization were analyzed with Native-PAGE and detected by GFP fluorescence (Fig. 3B). Each desired complex was observed as a major band, which was shifted to higher molecular weight positions as the number of sfGFP-ODNs increased. This result clearly shows that 2∼6 molecules of sfGFP-ODNs assemble at a very high yield. The hybridized product of six sfGFP-ODNs was directly observed by high-speed AFM (Fig. 3C). Six concatenated particles, each corresponding to sfGFP, were observed with various arrangements. Particles fluctuated during AFM imaging, reflecting their flexible nature (supporting information : Movie S1 and Movie S2).

**Figure 4:**
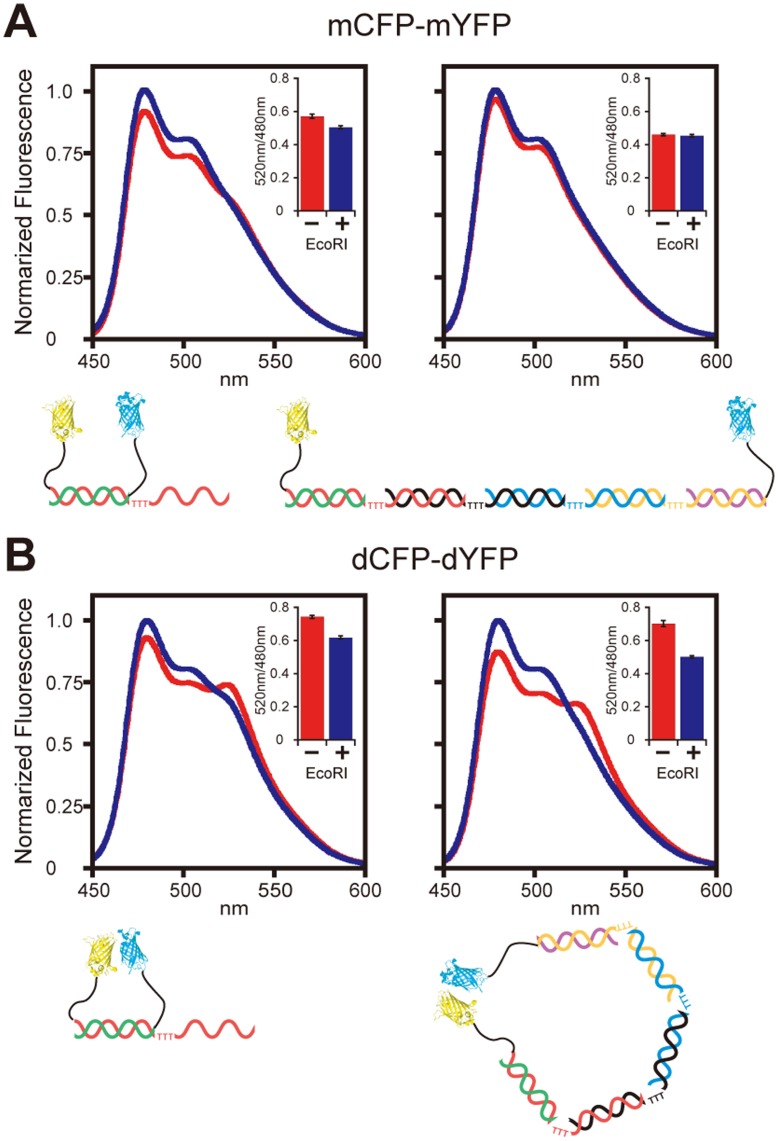
(A) Monomeric (A206K) and (B) dimeric (S208F/V224L) mutant of CFP and YFP were placed at adjacent position (left) and opposite ends (right). Fluorescence spectra were measured without (red) or with (blue) EcoRI treatment. Inset shows intensity ratio of 520 nm/480 nm as an indicator of FRET efficiency. The spectra are normalized to the value of 480 nm of the EcoRI-treated sample.

Finally, interactions between proteins aligned along the DNA backbone were demonstrated with FRET (fluorescent resonance energy transfer). We used two types of variants of GFP, cyan fluorescent protein (CFP) as a donor fluorescent protein and yellow fluorescent protein (YFP) as an acceptor fluorescent protein. It is known that the S208F/V224L mutant of GFP has a weak propensity to form a homo-dimer [20] while the A206K mutant exists as a stable monomer [21]. We introduced A206K and S208F/V224L mutations into CFP (termed mCFP and dCFP, respectively) and into YFP (termed mYFP and dYFP, respectively). They were connected to ODNs and assembled into multi-protein-DNA complexes in such a way that mCFP and mYFP (or dCFP and dYFP) were placed either at adjacent positions or at opposite ends. The fluorescence spectrum was measured and the ratio of fluorescent intensity of 520 nm/480 nm was calculated as an indicator of FRET from CFP to YFP (Fig. 4). As a control, the DNA backbone between CFP and YFP was cleaved by restriction enzyme EcoRI so that they could freely diffuse in the medium. When mCFP and mYFP were aligned in adjacent positions, the ratio of 520 nm/480 nm was decreased by EcoRI treatment, reflecting their physical vicinity in the multi-protein-DNA complex (Fig. 4A). No spectral change occurred before and after EcoRI treatment in the case where mCFP and mYFP were placed at opposite ends, indicating they are distant enough to abolish FRET. Combination of dCFP and dYFP, on the contrary, induced FRET even when they were placed at the end positions (Fig. 4B). The interaction between dCFP and dYFP is so weak that FRET disappeared once the DNA backbone was lost by EcoRI treatment. Obviously, increased local concentrations of dCFP and dYFP and flexibility of the hinge regions of the DNA backbone facilitate dimer formation with weak interactions.

## Discussion

The multi-protein-DNA complex reported here enables nano-scale alignment of several different proteins along a DNA backbone. Importantly, proteins are not tightly immobilized. Rather, they have some freedom of motion and can find the best orientation to interact with each other. Effective local concentrations of proteins are estimated to be ∼4.2 mM when six proteins are aligned in a DNA backbone and ∼1.4 mM when two proteins are aligned at positions on opposite ends (calculated from radius of gyration of freely-jointed chain model). These very high concentrations enable association of proteins with very weak association force. The nano-scale, flexible protein alignment has large potential for basic and application studies. Reaction cycles of multi-subunit complexes containing steps of subunit dissociation/association can occur more easily in the multi-protein-DNA complex.

## Supporting Information

Figure S1 **Mass spectroscopy analysis of the synthesized N_3_-ODN (A) 5′-aimino-ODN(No. 5) (17033Da) was detected as a peak at 17030.55 m/z.** (B) The synthesized N_3_-ODN(No. 5) (17306 Da) was detected as a peak at 17308.29 m/z. (C) Mass spectrum of the equal molar mixture of 5′-aimino-ODN and N_3_-ODN. The peaks of 5′-aimino-ODN (17033.28 m/z) and N_3_-ODN (17307.31 m/z) were indicated.
(TIF)

Figure S2 **Conjugation reaction of sfGFP and ODN was carried out using His6-sfGFP (without extra cysteine) and His6-sfGFP-Cys (with an extra cysteine) in the presence or absence of DBCO-PEG4-maleimide and N_3_-ODN.** The conjugated product was observed only for His6-sfGPF-Cys+DBCO-PEG4-maleimide+N_3_-ODN.
(TIF)

Figure S3 **Mass spectroscopy analysis of sfGFP-ODN.** (A) His6-sfGFP-Cys (27758Da) was detected as a peak at 27730.40 m/z. (B) His6-sfGFP-ODN (No. 5) (45739 Da) was detected as a peak at 45859.31 m/z.
(TIF)

Movie S1 **Movies of high-speed AFM of the multi-protein-DNA complex corresponding to the left panel (S1) and right panels (S2) of** **Fig. 3** **. Scan area, 80×80 nm^2^; time resolution, 145 msec/image.**
(MPG)

Movie S2 **Movies of high-speed AFM of the multi-protein-DNA complex corresponding to the left panel (S1) and right panels (S2) of** **Fig. 3** **. Scan area, 80×80 nm^2^; time resolution, 145 msec/image.**
(MPG)

Text S1 **Methods and results of Figure S1, S2 and S3.**
(DOCX)
