# Supplementary figures and images for: Nano-Scale Alignment of Proteins on a Flexible DNA Backbone
Source: PLoS One. 2012 Dec 26;7(12):e52534. doi: 10.1371/journal.pone.0052534 (PMC3530504; doi:10.1371/journal.pone.0052534)

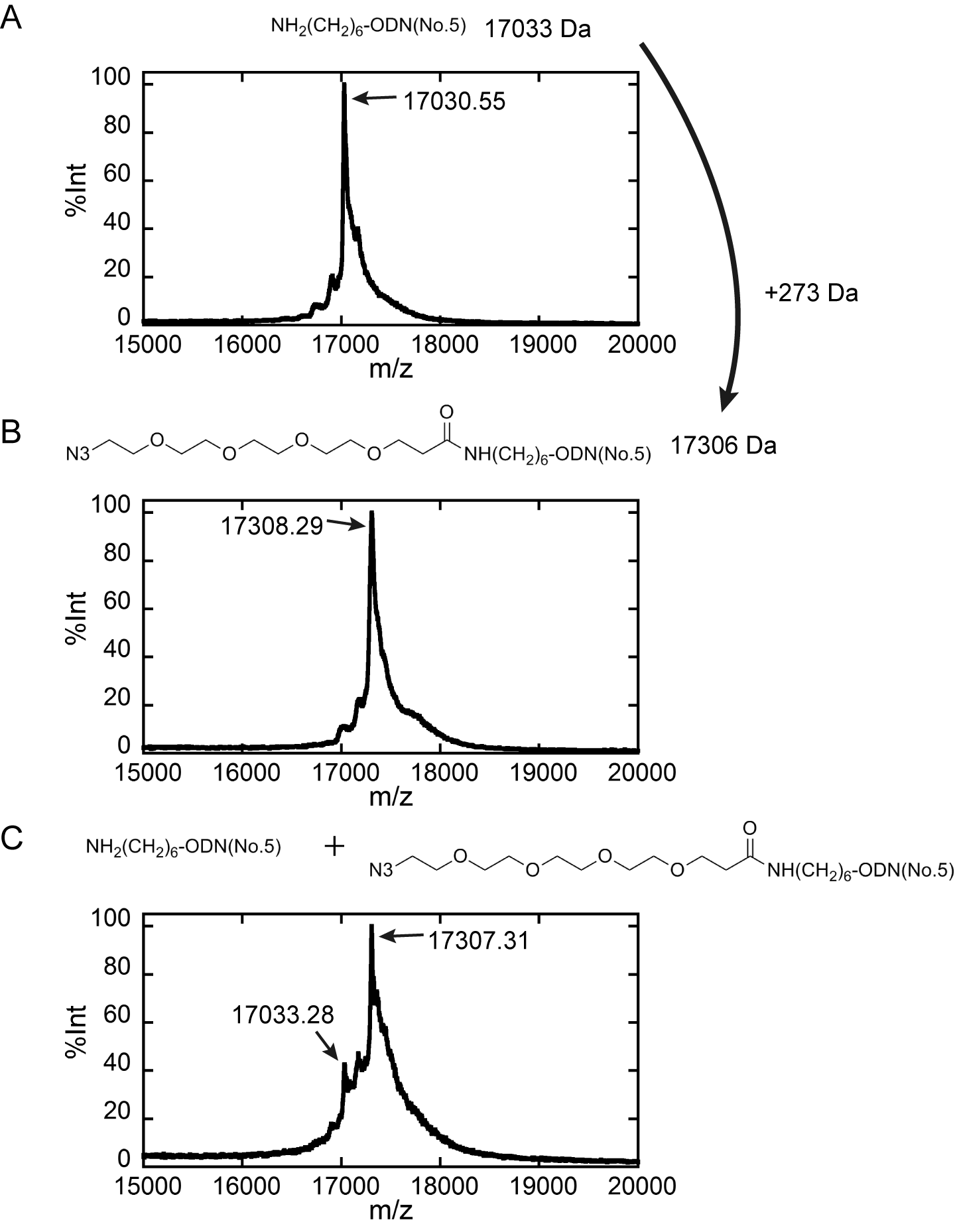

Supplement: Figure S1 — Mass spectroscopy analysis of the synthesized N3-ODN (A) 5′-aimino-ODN(No. 5) (17033Da) was detected as a peak at 17030.55 m/z. (B) The synthesized N3-ODN(No. 5) (17306 Da) was detected as a peak at 17308.29 m/z. (C) Mass spectrum of the equal molar mixture of 5′-aimino-ODN and N3-ODN. The peaks of 5′-aimino-ODN (17033.28 m/z) and N3-ODN (17307.31 m/z) were indicated. (TIF) [file pone.0052534.s001.tif]

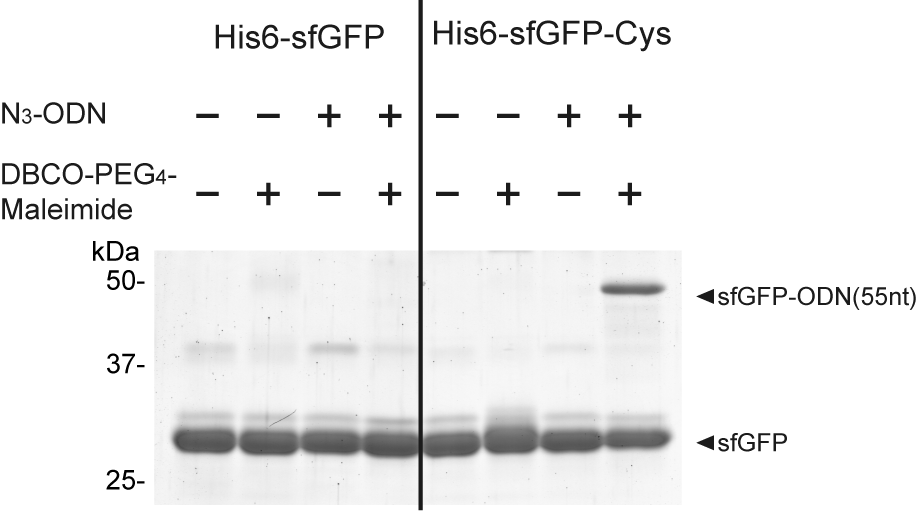

Supplement: Figure S2 — Conjugation reaction of sfGFP and ODN was carried out using His6-sfGFP (without extra cysteine) and His6-sfGFP-Cys (with an extra cysteine) in the presence or absence of DBCO-PEG4-maleimide and N3-ODN. The conjugated product was observed only for His6-sfGPF-Cys+DBCO-PEG4-maleimide+N3-ODN. (TIF) [file pone.0052534.s002.tif]

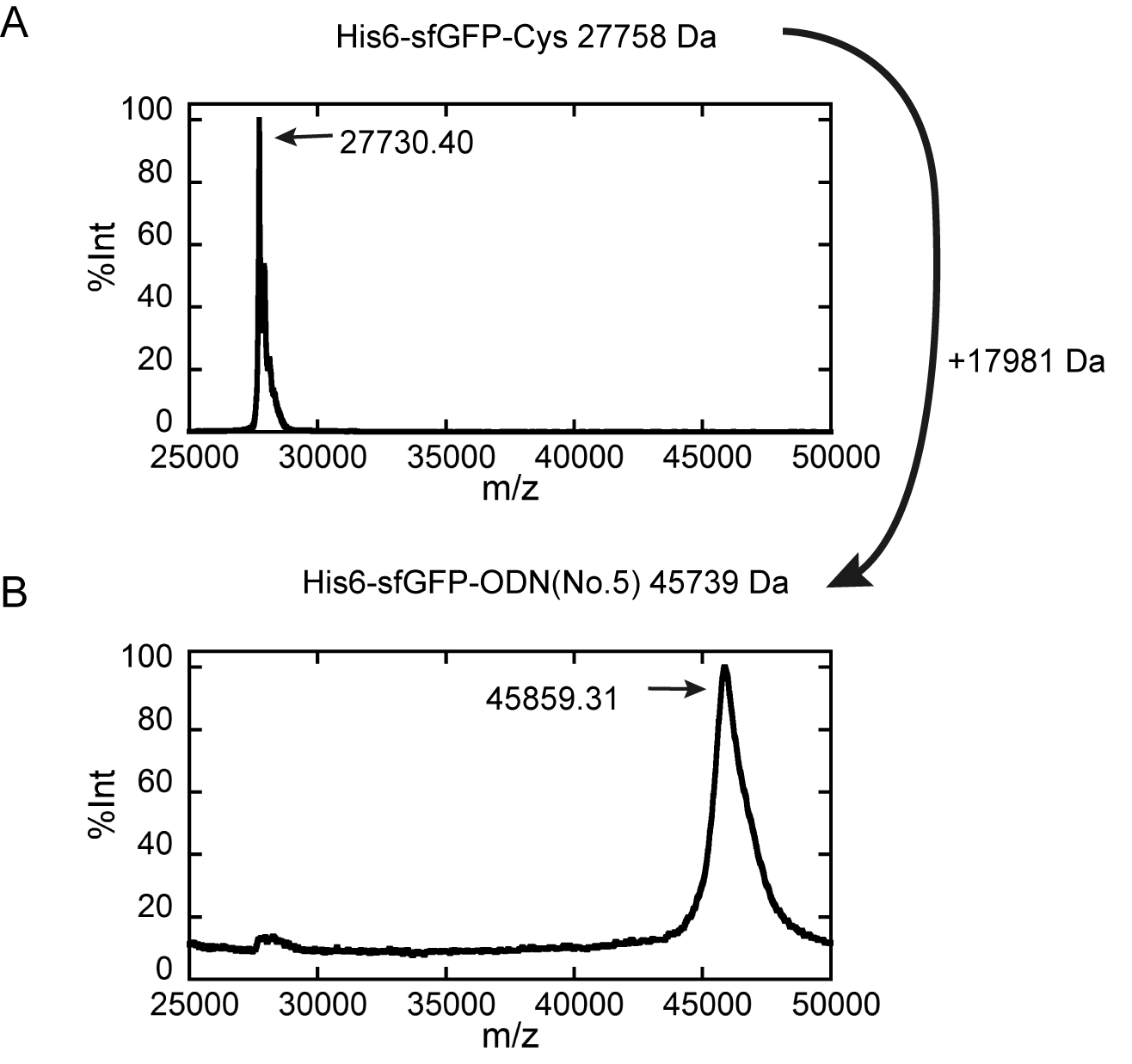

Supplement: Figure S3 — Mass spectroscopy analysis of sfGFP-ODN. (A) His6-sfGFP-Cys (27758Da) was detected as a peak at 27730.40 m/z. (B) His6-sfGFP-ODN (No. 5) (45739 Da) was detected as a peak at 45859.31 m/z. (TIF) [file pone.0052534.s003.tif]
