# Supplementary material for: Nano-Scale Alignment of Proteins on a Flexible DNA Backbone
Source: PLoS One. 2012 Dec 26;7(12):e52534. doi: 10.1371/journal.pone.0052534 (PMC3530504; doi:10.1371/journal.pone.0052534)
Supplement: Text S1 — Methods and results of Figure S1, S2 and S3. (DOCX) [file pone.0052534.s006.docx]

**PLOS One – Supporting Text**

**Full title**

Nano-scale alignment of proteins on a flexible DNA backbone

**Authors**

Tatsuya Nojima^1,*, †^, Hiroki Konno^2^, Noriyuki Kodera^2^, Kohji Seio^3^, Hideki Taguchi^4^, and Masasuke Yoshida^1^

**Methods**

**MALDI TOF MS analysis of N_3_-ODN**

After removal of unreacted azide-PEG4-NHS by anion-exchange column and desalting by ethanol precipitation, ODN (30 μM) was subjected to MALDI-TOF MS (AXIMA-CFR Plus, Shimadzu) in linear mode using 3-hydroxypicolinic acid matrix.

**MALDI TOF MS analysis of sfGFP-ODN**

Purified His_6_-sfGFP-Cys and His_6_-sfGFP-ODN was analyzed by MALDI-TOF MS. Samples were dialyzed against pure water and lyophilized. Lyophilized samples were dissolved in pure water to the concentration of about 25 μM and analyzed by MALDI-TOF MS in linear mode. Sinapic acid matrix was used for His_6_-sfGFP-Cys and 3-hydroxypicolinic acid was used for His_6_-sfGFP-ODN.

**Result**

**Mass spectroscopy of synthesized N_3_-ODN**

Synthesized N_3_-ODN was analyzed by mass spectroscopy (Fig. S1). The calculated molecular weight of N_3_-ODN is 273 Da larger than 5’-aimino-ODN. 5’-aimino-ODN(No. 5) (Fig. S1A) and the synthesized N_3_-ODN(No. 5) (Fig. S1B) were analyzed by MALDI-TOF MS and their corresponding m/z peaks were detected. When an equal molar mixture of 5’-aimino-ODN and N_3_-ODN was analyzed by MALDI-TOF MS (Fig. S1C), the peak height of 5’-aimino-ODN was ~40% of that of N_3_-ODN, indicating that ionization efficiency of 5’-aimino-ODN was lower than N_3_-ODN. However, even if this low ionization efficiency of 5’-aimino-ODN is taken into account, spectrum in Fig. S1B suggests that N_3_-ODN was mostly pure.

**Structure of sfGFP-ODN**

The structure of sfGFP-ODN was confirmed by the following experiments. We introduced an extra cysteine residue at the C-terminus of His_6_-sfGFP (His_6_-sfGFP-Cys) for conjugation with N_3_-ODN using DBCO-PEG_4_-Maleimide as a cross-linker. To confirm that ODN was conjugated through the cysteine, we carried out the same conjugate reaction using His_6_-sfGFP without an extra cysteine residue (Fig. S2). Conjugated sfGFP-ODN was formed only when His_6_-sfGFP-Cys was reacted in the presence of the cross-linker. Next, mass spectroscopy analysis of sfGFP-ODN was carried out (Fig. S3). After conjugation, the molecular weight should increase by 17981 Da from 27758 Da of His_6_-sfGFP-Cys to 45739 Da of His_6_-sfGFP-ODN. Indeed, the corresponding m/z peak was detected by MALDI-TOF MS. These results clearly show that sfGFP and ODN were conjugated as designed.
